# Air pollution and adult hospital admissions for ischemic stroke: a time-series analysis in Inner Mongolia, China

**DOI:** 10.1265/ehpm.24-00311

**Published:** 2025-04-26

**Authors:** Sen Feng, Chunhua Li, Yujing Jin, Haibo Wang, Ruying Wang, Zakaria Ahmed Mohamed, Yulong Zhang, Yan Yao

**Affiliations:** 1 Epidemiology and Biostatistics, School of Public Health, No. 1163, Xinmin Street, Jilin University, Changchun 130021, Jilin, China; 2 Department of Endocrinology, Affiliated Hospital of Changchun University of Chinese Medicine, Changchun 130012, Jilin, China; 3 Department of Developmental and Behavioral Pediatrics, The First Hospital of Jilin University, Changchun 130021, Jilin, China; 4 Department of Laboratory Medicine, Xing’an League People’s Hospital, 137400, Inner Mongolia, China

**Keywords:** Air pollutant, Ischemic stroke, Cumulative effect

## Abstract

**Background:**

Previous studies have demonstrated that short-term exposure to ambient particulate matter elevates the risk of ischemic stroke in major urban areas of various countries. However, there is a notable gap in research focusing on remote areas inhabited by ethnic minorities and the cumulative effects of air pollutants. Our study conducted in the area aims to explore the potential association between ischemic stroke and air pollutants and contribute to improving health outcomes among the community.

**Methods:**

This retrospective observational study was conducted at the Xing’an League People’s Hospital in Inner Mongolia. The medical records of 4,288 patients admitted for IS between November 1, 2019, and October 31, 2020, were reviewed. Data on demographics (age and sex), air pollutants (PM_10_, PM_2.5_, NO_2_, NO, CO, and O_3_), and meteorological factors (daily average temperature, daily average wind speed, and daily average atmosphere pressure) were collected and analyzed. The statistical analysis included descriptive statistics, Poisson distribution analysis to evaluate the adverse effects of atmospheric pollutants on daily hospitalizations, and subgroup analysis to determine whether gender and age could modify the impact on hospitalizations.

**Results:**

A substantial correlation was revealed in single-day lags model. The peak delayed effects of PM_10_, PM_2.5_, SO_2_, and NO_2_ were observed at lag8 (PM_10_ (OR = 1.016, 95%CI 1.002, 1.030), PM_2.5_ (OR = 1.027, 95%CI 1.007, 1.048), SO_2_ (OR = 1.153, 95%CI 1.040, 279) and NO_2_ (OR = 1.054, 95%CI 1.005, 1.105)) while males exhibited a consistent trend from lag0 to lag8 (PM_10_ (OR = 1.035, 95%CI 1.018, 1.053), PM_2.5_ (OR = 1.056, 95%CI 1.030, 1.082), SO_2_ (OR = 1.220, 95%CI 1.072, 1.389), NO_2_ (OR = 1.126, 95%CI 1.061, 1.120), CO (OR = 10.059, 95%CI 1.697, 59.638) and O_3_ (OR = 0.972, 95%CI 0.946, 0.999)). When gender and age were considered, a positive impact was also observed after three days cumulative effect in males.

**Conclusions:**

There is a significant cumulative effect of exposure to air pollution on IS hospital admissions, especially the males and patients under the age of 65. Our results also suggested that a notable association between CO and NO_2_ in two-pollutant models.

## 1. Introduction

Stroke is the most common serious manifestation of cerebrovascular disease, ranking as the fifth leading cause of death in the United States [1] and the second leading cause of death globally [2]. It is typically categorized into two main types: ischemic and hemorrhagic, with ischemic stroke (IS) accounting for 87% of cases [3]. IS is characterized by acute brain cell damage resulting from reduced blood flow to the brain. Approximately 700,000 individuals suffer an IS in the US each year [4]. According to the latest evidence from the GBD (Global Burden of Disease) study, ambient air pollution is recognized as the primary environmental risk factor for global mortality and morbidity. In 2015, it was estimated that ambient air pollution caused 4.2 million deaths, accounting for 7.6% of total global mortality, and resulted in 103.1 million disability-adjusted life years (DALYs), which represents 7.6% of global DALYs [5]. Additionally, it is also considered as a risk factor for stroke [6] and studies have shown that ambient air pollution is more strongly associated with stroke incidence compared to indoor air pollution [7]. Previous studies have shown a significant relationship between gaseous pollutants, particulate matter, and stroke hospitalization rates [8]. However, these studies have primarily focused on large cities or top-ranked hospitals in China [9–11]. Xing’an League People’s Hospital is situated in Ulanhot City, within the Xing’an League of Inner Mongolia. As the sole comprehensive tertiary hospital in the league, it provides medical and health services to a population of 2 million residents in the league and its surrounding areas, and it is equipped with 1,460 beds. In 2023, the hospital recorded over 854,500 outpatient and emergency visits, as well as 62,900 patient discharges. Numerous studies have indicated that the investigation of the association between hospitalization for IS and atmospheric pollutant is hindered by geographical factors and climatic conditions. To the best of our knowledge, there has been no study conducted on the relationship between air pollution and IS in Inner Mongolia Province. Inner Mongolia, located in northern China, is a vast territory characterized by a unique plateau climate due to its high latitude. This region is home to various ethnic minorities. With a population of approximately 24 million, Inner Mongolia experiences both a temperate continental climate and a temperate monsoon climate, covering a total area of 1.183 million square kilometers, making it the third largest province in China. By the end of 2023, the permanent population is projected to be 23.960 million, with a regional GDP of 2.4627 billion yuan and a per capita GDP reaching 100,000 yuan [12]. Among the nine provinces in the northern and western regions of China (Heilongjiang, Tibet, Jilin, Liaoning, Xinjiang, Hebei, Inner Mongolia, Beijing, and Ningxia), the Inner Mongolia Autonomous Region ranks seventh in the incidence of IS [13]. In comparison to other cities in China, the Inner Mongolia Autonomous Region experiences high levels of air pollution [14]. Air pollutants have the potential to impact the risk of IS and exhibit time lag characteristics. Therefore, studying the association between ambient air pollution and the occurrence of IS in Inner Mongolia holds great significance, especially considering the high proportion of ethnic minorities and relatively low air pollution concentrations among the population.

## 2. Materials and methods

### 2.1 Study design and population

This was a retrospective observational study conducted at Xing’an League People’s Hospital, Inner Mongolia. The medical records of 4288 patients admitted for IS between Nov. 1 2019 to Oct. 31 2020 were reviewed. Patients over the age of 18 who were admitted to the inpatient department of our hospital with a diagnosis of IS (I63.0–I63.9) were included in this study. Stroke was diagnosed by using the International Classification of Diseases (ICD-10). We categorized the patients into two age groups: (a) 64 years and younger and (b) 65 years and older, taking into account both genders.

### 2.2 Data collection

Data were collected from the Xing’an League People’s Hospital’s electronic data database. Data is collected and managed by Xing’an League People’s Hospital in strict accordance with established rules and regulations. The hospital emphasizes the importance of privacy protection and ethical principles regarding patient confidentiality and data security. The data is characterized by its comprehensiveness, accuracy, compliance, and confidentiality, and is typically maintained in electronic form, which is managed, stored, and encrypted. The collected data comprised demographic characteristics of patients, air pollution, and meteorological data. Demographic data includes age, gender, diagnosis, admission time, address, and other relevant information. Air pollution and meteorological data came from China Meteorological Data Service Network (http://data.cma.cn/), including daily average temperature (temperature, temp, °C) and average wind speed (wind speed, ws, km/h). The automatic observation project of the weather station conducts 24 regular observations a day and takes the average value. Real-time hourly air quality data including (PM_10_, PM_2.5_, NO_2_, NO, CO, and O_3_) for the Hinggan League from November 1, 2019, to October 31, 2020, were collected from monitoring stations operated by the Xing’an League Meteorological Bureau (46°08′N, 122°05′E, 2 kilometers away from Xing’an League People’s Hospital). The region includes a total of 392 air quality monitoring stations and 9 meteorological stations. However, due to the geographical concentration of study participants in the Xing’an League area, we primarily relied on data from the local monitoring station to ensure spatial consistency with hospital admissions. Missing data were supplemented by data collected at other weather stations located near Hinggan League. The Xing’an League Meteorological Bureau validated the data using the Quality Control of Surface Meteorological Observation Data.

### 2.3 Statistical analysis

Across an entire resident population, incidences of admission are random events occurring on any particular day that can be described by a Poisson distribution. Due to the nonlinear relationship between admission and other variables, a time-series analysis was conducted using generalized additive models to estimate the correlations between air pollutants and mortality, thus,

$$\begin{array}{rl} \log[E(Y_{\text{t}})] & =\alpha +\beta \times X_{\text{t}}+\text{as.factor(dow)}\\ & \;+\text{as.factor(holiday)}+s(t,\mathrm{df})+s(Z_{\text{t}},\mathrm{df}) \end{array}$$

where *Y_t_* is the total daily admissions for IS on day t, *E*(*Y_k_*) is the expected value of *Y_t_*, *β* is the regression coefficient of a specific pollutant *X* on day *t* (the exposure-response relationship coefficient), *s* is the non-parametric spline smoothing function, *df* is the degrees of freedom, *dow* represents the weekday dummy variable, while *holiday* is a binary variable representing whether day *t* is a public holiday or not, and seasonal dummy variables, *t* is the calendar time, *Z_t_* represents meteorological factors such as average temperature, average wind speed and sea level pressure on day *k*, and *α* is the residual value.

Poisson regression provides an estimation of relative risk as RR = exp. (*β*) where *β* is the regression coefficient with a unit increment in an air pollutant, we chose 15 lag days for our models in the current study. Inter quartile range (IQR) was used as increment of every pollutant to calculate the cumulative effect on daily respiratory disease admissions with lag days of 0–15. Relationship analysis of air pollution and hospital admission was conducted for genders (male and female), age groups (<64 years, and ≥65 years), and different seasons (warm season: May to November; cold season: January to April and December).

Results were presented in excess risk (ER), the percentage increase for IQR exposure increments, which was derived from (RR − 1) × 100%. Positive ER values indicated the percentage increase (%) in respiratory hospital admissions for an unit level increase in pollutant concentration. Negative ER values indicate there is a negative effect or no relationship between air pollutant and hospital admissions. All statistical analyses were conducted by using R (version 4.2.3, http://www.r-project.org) packages.

#### 2.3.1 Single-pollutant model

Across an entire resident population, incidences of admission are random events occurring on any particular day that can be described by a Poisson distribution. Because the relationship between admission and other variables is nonlinear, the time-series analysis was applied using generalized additive Poisson regression models to estimate the correlations between air pollutants and admission, thus,

$$\begin{array}{rl} \log[E(Y_{t})] & =\alpha +\beta \times X_{t}+\text{as.factor}(\text{dow})\\ & \;+\text{as.factor}(\text{holiday})+s(t,\mathrm{df})+s(Z_{t},\mathrm{df}) \end{array}$$

We also assessed the sensitivity of the smoothing function to time trends and meteorological factors. The holiday variable was introduced to control for systematic variation over time. Air pollutants were added separately into lag models. We separately tested PM_10_, PM_2.5_, NO_2_, NO, CO and O_3_ using multiday lags (lag0–1, lag0–2, lag0–3, lag0–4, lag0–5, lag0–6, lag0–7, lag0–8, lag0–9, lag0–10, lag0–11, lag0–12, lag0–13, lag0–14, and lag0–15), and excess risk (ER) was applied using the units of concentration increases to determine the correlation between the air pollutants and IS admission.

#### 2.3.2 Two-pollutant model

Two-pollutant models were applied to examine the independent effects of the pollutants. We used two-pollutant adjusted models with the lag structure to determine the sensitivity of correlation between one pollutant and IS admission after adjusting for the other pollutants. For instance, we controlled the concentration of CO in the model to further analyze the effects of PM_2.5_ on IS admission without the confounding effect of CO. The model was as follows:

$$\begin{array}{rl} \log[E(Y_{t})] & =\alpha +\beta \times X_{\text{PM2.5}}+\beta \times X_{\text{CO}}\\ & \;+\text{as.factor}(\text{dow})+\text{as.factor}(\text{holiday})\\ & \;+s(t,\mathrm{df})+s(Z_{t},\mathrm{df}) \end{array}$$

where *X_PM2.5_* is the concentration of PM_2.5_, and *X_CO_* is the concentration of CO.

Hence, collinearity among pollutants was taken into account in this study.

All analyses were performed using the “mgcv” package in R-4.2.3 statistical software.

## 3. Results

### 3.1 Descriptive statistics

Table 1 presents descriptive statistics of the study population, meteorological data, and air pollution data. There were 4288 incidences of admission by IS during the study period. The total population consists of 62.13% males and 37.87% females; according to age stratification, the number of residents under 64 age group who were admitted to hospital due to IS is more than the number of residents over 65 who were admitted to hospital due to IS; 56.53% of residents under 64 age group and 43.47% residents over 65. IS admission per day was higher in males than females (7.27 ± 3.57 for males compared with 4.45 ± 2.71 for females) and was higher for people aged from 18 to 64 (1.96 ± 1.15) compared with people aged 64 or above (1.18 ± 0.61) and people aged 18 or below (0.01 ± 0.10). Daily mean concentrations for PM_10_, PM_2.5_, SO_2_, NO_2_, CO and O_3_ were 25.49 µg/m^3^, 39.31 µg/m^3^, 5.57 µg/m^3^, 14.20 µg/m^3^, 0.43 mg/m^3^, and 56.25 µg/m^3^. The O_3_ concentration in the meteorological data is the daily maximum 8 h average, and other items are 24 h averages. The number of meteorological days included in the study was 366 days. The study period included 366 days of pollutant-related data. Most daily averages of PM_10_, PM_2.5_, SO_2_, NO_2_, CO and O_3_ are all lower than the national secondary air quality standard.

**Table 1:** Distribution characteristics of hospitalization, meteorological factors and air pollutants in Hinggan League, China, 2019–2020

**Characteristic**	**n (percent)**	**Mean (SD)**	**Minimum**	**P25**	**P50**	**P75**	**Maximum**	**IQR**
CIS								
Total patients	4288 (100)	11.71 (5.01)	1.00	8.00	11.00	15.00	30.00	7.00
Male	2664 (62.13)	7.27 (3.57)	0	5.00	7.00	9.00	21.00	4.00
Female	1624 (37.87)	4.45 (2.71)	0	2.00	4.00	6.00	14.00	4.00
Age								
0–17	4 (0.09)	0.01 (0.10)	0	0	0	0	1	0
18–64	2420 (56.44)	6.61 (3.20)	0	4.00	6.00	9.00	19.00	5.00
≥65	1864 (43.47)	5.09 (2.77)	0	3.00	5.00	7.00	15.00	4.00
Season								
Warm season	2482 (57.88)	11.60 (4.66)	1.00	8.00	11.00	14.00	28.00	6.00
Cold season	1806 (42.12)	11.88 (5.48)	1.00	8.00	11.00	15.00	30.00	7.00
Air pollutions (µg/m^3^)								
PM_2.5_	/	25.49 (17.73)	9.00	14.00	19.00	30.00	109.00	16.00
PM_10_	/	39.31 (23.91)	11.00	23.00	32.00	48.00	152.00	25.00
SO_2_	/	5.57 (3.84)	2.00	3.00	4.00	7.00	28.00	4.00
NO_2_	/	14.20 (6.90)	3.00	9.00	13.00	18.00	38.00	9.00
CO	/	0.43 (0.25)	0.10	0.25	0.35	0.54	1.630	0.290
O_3_	/	56.25 (20.01)	16.00	41.00	55.50	69.0	137.00	28.00
Meteorological factors								
Temperature, °C	/	8.71 (13.29)	−17.70	−4.13	10.30	20.45	29.80	24.58
Wind speed, km/h	/	8.80 (3.04)	3.50	6.50	8.30	10.60	21.30	4.10
Atmosphere pressure, Hpa	/	969.02 (69.74)	946.05	959.57	966.17	972.50	987.68	12.93
Relative humidity, %	/	59.36 (223.93)	18.63	49.21	60.68	71.03	90.02	21.82

Note: SD, standard deviation. *According to the Chinese Ambient Air Quality Standards GB3095-2012 (CAAQS-GB3095-2012), it is recommended to take the short-term (24-h) AQG level of CO, NO_2_, PM_10_, PM_2.5_ and SO_2_ as the living area target, and it is recommended to adopt 4 mg/m^3^, 80 µg/m^3^, 150 µg/m^3^, 75 µg/m^3^, 150 µg/m^3^, respectively. The average of daily maximum 8-h mean concentrations of 160 µg/m^3^ is represented ozone’s standard.

**Fig. 1:**
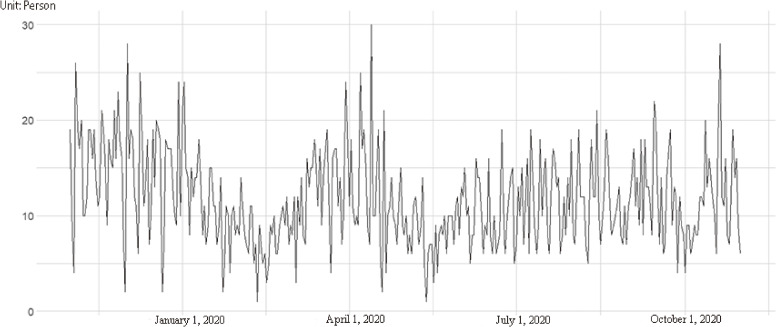
The temporal pattern of daily admissions of ischemic stroke

Figure 1 shows the dynamic chart of daily admissions of ischemic stroke patients at Xing’an League People’s Hospital during the study period.

### 3.2 Spearman’s rank correlation analysis

Table 2 presents the relationships between the date, air pollutant concentrations, and meteorological variables. Most pollutants were significantly positively correlated with each other and negatively correlated with temperature (*R^2^* values for daily average temperature against PM_2.5_, PM_10_, SO_2_, NO_2_ and CO were −0.440, −0.316, −0.686, −0.120 and −0.430, respectively). The levels of PM_10_, PM_2.5_, SO_2_, NO_2_, and CO were positively correlated with each other (correlation coefficient = 0.427–0.891, P < 0.05), while O_3_ was negatively correlated with other air pollutants but NO_2_ and CO (Table 2).

**Table 2:** Spearman correlation coefficients among the exposure variables in Hinggan League, 2019–2020

**Variables**	**PM_2.5_**	**PM_10_**	**SO_2_**	**NO_2_**	**CO**	**O_3_**	**Temp**
PM_2.5_	1.00	0.891**	0.680**	0.621**	0.830**	−0.031	−0.440**
PM_10_	—	1.00	0.616**	0.662**	0.752**	0.010	−0.316**
SO_2_	—	—	1.00	0.427**	0.758**	−0.084	−0.686**
NO_2_	—	—	—	1.00	0.694**	−0.353**	−0.120*
CO	—	—	—	—	1.00	−0.112*	−0.430**
O_3_	—	—	—	—	—	1.00	0.322**
Temp	—	—	—	—	—	—	1.00

**P < 0.001

*P < 0.05

Abbreviations: PM_2.5_, particulate matter ≤2.5 µm in aerodynamic diameter; PM_10_, particulate matter ≤10 µm in aerodynamic diameter; Temp, temperature; RH, relative humidity.

### 3.3 Single-pollutant models

**Fig. 2:**
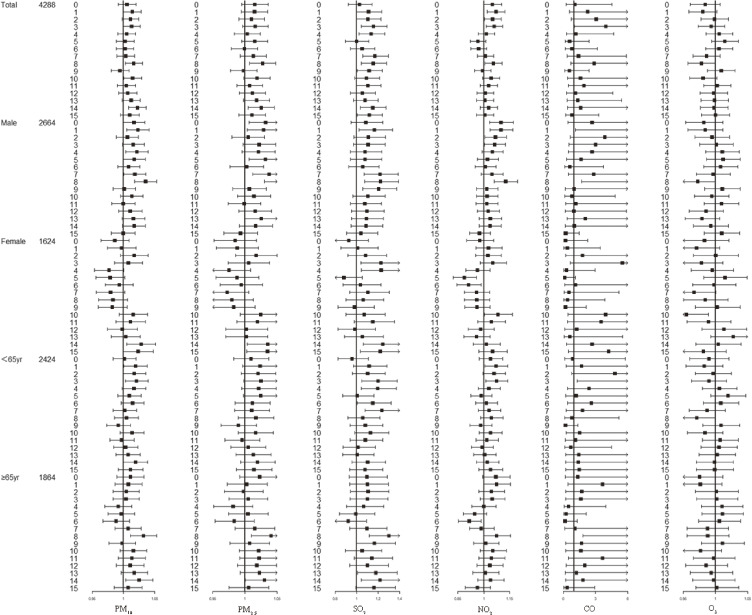
Forest plot in lags model of all IS admissions by total, sex and age

After adjusting for long-term trends, holiday effects, temperature and relative humidity, Fig. 2 displays the results for single-day lags 0–15 of PM_10_, PM_2.5_, SO_2_, NO_2_, CO, and O_3_, categorized by gender and age. The peak delayed effects of PM_10_, PM_2.5_, SO_2_, and NO_2_ were observed at lag8 (PM_10_ (OR = 1.016, 95%CI 1.002, 1.030), PM_2.5_ (OR = 1.027, 95%CI 1.007, 1.048), SO_2_ (OR = 1.153, 95%CI 1.040, 279) and NO_2_ (OR = 1.054, 95%CI 1.005, 1.105)) while PM_10_, PM_2.5_ and SO_2_ also showed effects again at lag14, PM_10_ (OR = 1.022, 95%CI 1.008, 1.036), PM_2.5_ (OR = 1.025, 95% CI 1.005, 1.046) and SO_2_ (OR = 1.149, 95%CI 1.035, 240). When gender and age were considered, males exhibited a consistent trend from lag0 to lag8 (PM_10_ (OR = 1.035, 95%CI 1.018, 1.053), PM_2.5_ (OR = 1.056, 95%CI 1.030, 1.082), SO_2_ (OR = 1.220, 95%CI 1.072, 1.389), NO_2_ (OR = 1.126, 95%CI 1.061, 1.120), CO (OR = 10.059, 95%CI 1.697, 59.638) and O_3_ (OR = 0.972, 95%CI 0.946, 0.999)) compared to the total population. The study demonstrated statistical significance from lag0 to lag4, for certain pollutants, while women consistently showed a significant association with total lag14 for PM_10_ (OR = 1.028, 95%CI 1.005, 1.051), PM_2.5_ (OR = 1.035, 95%CI 1.004, 1.068), SO_2_ (OR = 1.244, 95%CI 1.058, 1.461). The Fig. 2 indicated that CO and O_3_ did not have a significant impact on the admission rate of IS patients in the overall population and age subgroups. However, a positive association was observed at lag8 in the male group.

**Fig. 3:**
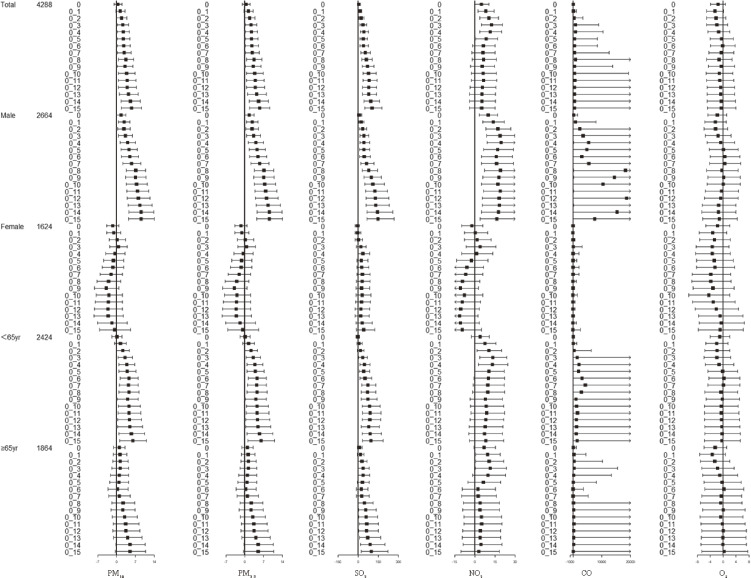
Forest plot in multiday lags of all IS admissions by total, sex and age

Figure 3 summarizes the results of the subgroup analysis of all IS admissions by sex and age, with cumulative lag days ranging from 0 to 15. A significant positive correlation was found between total IS admissions and concentrations of PM_10_, PM_2.5_, and SO_2_. However, NO_2_ showed a significant positive correlation with total IS admissions only for lag days 1–5. The association between PM_10_, PM_2.5_, SO_2_, NO_2_, CO, and daily IS admissions was significant in male groups. Further stratification analysis revealed that males had a stronger association with patient admissions compared to other subgroups. Notably, males showed a significant and stronger association with all atmospheric pollutants. Specifically, NO_2_ exhibited a significant association in the male subgroup. When stratified by age, both subgroups displayed a similar trend to the total population. However, IS patients under the age of 65 were found to be more susceptible to the impact of air pollutants, leading to hospital admissions.

### 3.4 Two-pollutant models

Table 3 presents the estimated values of the influential lag effects for the two-pollutant models. After adjusting for another five air pollutants, the irrelevant effects of PM_10_ and SO_2_ on IS were the most stable across all two-pollutant analyses. However, for PM_2.5_ and NO_2_, the associations were attenuated and became significant with the addition of CO. Remarkably, with the addition of NO_2_, which was negatively correlated with CO, the estimated ERs of CO decreased and obtained statistical significance.

**Table 3:** Odds ratio for combined effects of air pollutants on daily IS admissions*

**Variable**	**PM_2.5_**	**PM_10_**	**SO_2_**	**NO_2_**	**CO**	**O_3_**
Adjusted for PM_2.5_	—	0.97 (0.94,1.01)	0.99 (0.87,1.12)	1.04 (0.98,1.12)	0.85 (0.69,1.06)	0.98 (0.96,1.01)
Adjusted for PM_10_	1.05 (1.00,1.11)	—	1.01 (0.89,1.14)	1.06 (0.99,1.13)	0.94 (0.77,1.15)	0.98 (0.96,1.01)
Adjusted for SO_2_	1.02 (0.99,1.04)	1.00 (0.99,1.02)	—	1.06 (1.00,1.12)	0.98 (0.82,1.17)	0.98 (0.96,1.08)
Adjusted for NO_2_	1.00 (0.98,1.03)	0.99 (0.98,1.01)	0.97 (0.86,1.10)	—	0.76 (0.59,0.97)	0.99 (0.97,1.02)
Adjusted for CO	1.03 (1.00,1.06)	1.00 (0.00,1.03)	1.03 (0.90,1.18)	1.13 (1.04,1.23)	—	0.98 (0.96,1.01)
Adjusted for O_3_	1.01 (0.99,1.04)	1.01 (0.99,1.02)	1.02 (0.91,1.14)	1.04 (0.99,1.10)	0.98 (0.85,1.14)	—

*Two-pollutant models of PM_10_ and PM_2.5_ are difficult to interpret because of high correlation between PM_10_ and PM_2.5_.

Table 4–5 examines the subgroups of two-pollutant models across different genders. In the male group, the adjusted model results for PM_2.5_ and NO_2_ are generally significant, whereas the female group shows no significant findings.

**Table 4:** Odds ratio for combined effects of air pollutants on daily IS admissions in males*

**Variable**	**PM_2.5_**	**PM_10_**	**SO_2_**	**NO_2_**	**CO**	**O_3_**
Adjusted for PM_2.5_	—	0.98 (0.93,1.02)	1.01 (0.87,1.18)	1.08 (0.99,1.17)	0.25 (0.02,3.89)	0.98 (0.96,1.01)
Adjusted for PM_10_	1.06 (0.99,1.14)	—	1.03 (0.88,1.20)	**1.10** **(1.01,1.19)**	0.74 (0.57,9.60)	0.98 (0.95,1.01)
Adjusted for SO_2_	**1.03** **(1.00,1.06)**	1.01 (0.99,1.03)	—	**1.10** **(1.03,1.18)**	0.70 (0.19,15.32)	0.98 (0.96,1.01)
Adjusted for NO_2_	1.01 (0.98,1.05)	1.00 (0.98,1.02)	0.99 (0.84,1.16)	—	0.05 (0.00,1.15)	0.99 (0.96,1.03)
Adjusted for CO	**1.05** **(1.01,1.09)**	1.02 (0.99,1.04)	1.06 (0.90,1.25)	**1.19** **(1.07,1.32)**	—	0.98 (0.95,1.01)
Adjusted for O_3_	**1.03** **(1.01,1.06)**	1.02 (0.99,1.03)	1.08 (0.94,1.23)	**1.10** **(1.02,1.17)**	2.21 (0.35,13.75)	—

*Two-pollutant models of PM_10_ and PM_2.5_ are difficult to interpret because of high correlation between PM_10_ and PM_2.5_.

**Table 5:** Odds ratio for combined effects of air pollutants on daily IS admissions in females*

**Variable**	**PM_2.5_**	**PM_10_**	**SO_2_**	**NO_2_**	**CO**	**O_3_**
Adjusted for PM_2.5_	—	0.97 (0.92,1.03)	0.95 (0.78,1.16)	1.00 (0.90,1.11)	0.19 (0.01,6.90)	0.98 (0.94,1.02)
Adjusted for PM_10_	1.02 (0.94,1.11)	—	0.97 (0.79,1.19)	1.01 (0.91,1.12)	0.35 (0.01,10.01)	0.98 (0.94,1.02)
Adjusted for SO_2_	0.99 (0.95,1.03)	0.99 (0.96,1.01)	—	0.99 (0.90,1.08)	0.24 (0.01,4.25)	0.98 (0.94,1.02)
Adjusted for NO_2_	0.98 (0.94,1.03)	0.98 (0.95,1.01)	0.93 (0.76,1.15)	—	0.07 (0.00,3.70)	0.98 (0.94,1.02)
Adjusted for CO	1.00 (0.95,1.06)	0.99 (0.96,1.03)	0.98 (0.79,1.23)	1.05 (0.92,1.20)	—	0.97 (0.94,1.02)
Adjusted for O_3_	0.98 (0.95,1.02)	0.98 (0.96,1.01)	0.92 (0.77,1.10)	0.96 (0.88,1.04)	1.17 (0.02,1.82)	—

*Two-pollutant models of PM_10_ and PM_2.5_ are difficult to interpret because of high correlation between PM_10_ and PM_2.5_.

## 4. Discussion

This study represents the first comprehensive examination of the association between exposure to ambient air pollution and IS in Inner Mongolia. Our findings indicate that, overall, the pollutants studied—except for O_3_—had a significant impact on IS in this region, particularly emphasizing the cumulative effect. Furthermore, subgroup analyses revealed that the toxic effects were more pronounced in males and among individuals aged 65 years and younger. The two-pollutant model indicated that, after adjusting for NO_2_, the influence of CO appeared to be protective. What causes the inconceivable effect of CO remains to be explored.

In Fig. 1, the daily number of admissions to IS declined from January to April 2020. This decline can be attributed not only to the effects of cold weather but also to the reporting of the first confirmed case of COVID-19 in the Inner Mongolia Xing’an League on January 26, 2020, and the subsequent activation of emergency management measures, which may have indirectly contributed to the reduction in hospital admissions.

In single-pollutant model that did not consider time effects, the concentration of air pollutants had no significant impact on the number of hospitalizations for IS. Previous researches also had yielded inconclusive findings on this matter. For instance, Gregory A found that PM_2.5_ concentrations were linked to an increased risk of IS [15]. A study by Tian and colleagues across 172 cities in China indicated that all air pollutants analyzed, except CO and O_3_, were significantly associated with higher hospital admissions for IS in a single pollutant model [16]. However, some studies suggest there is no significant correlation between air pollutants and stroke. For example, a study in Tianjin (Cui, Y et al.) found no clear associations between gaseous pollutants and the number of hospitalized patients with IS in most cases [17]. Some studies have identified a complex U-shaped relationship between O_3_ levels and ischemic stroke in certain diseases, such as type 2 diabetes [18]. This complexity may arise from the difficulty in distinguishing between environmental pollution and multifaceted air pollution. Our investigation found no evidence of an association between exposure to O_3_ and CO and the risk of hospital admissions for total stroke or ischemic stroke [19]. These findings are consistent with a study that utilized data from the Women’s Health Initiative, which similarly did not observe any associations between daily exposure to O_3_ and CO and stroke or its subtypes [20]. When considered with the lag effect of time, PM_10_, PM_2.5_, SO_2_, and NO_2_ all showed obvious adverse effects on lag8. In contrast to other studies that examine short-term effects and report significant lag effects over a duration of 3–4 days, our findings indicate a distinct delay in the lag effect, differing from previous research conducted in developed or high-income regions. As an underdeveloped region, Inner Mongolia exhibits significant differences in economic, cultural, and geographical factors compared to high-income areas. This disparity may contribute to the observed phenomenon, as individuals in high-income brackets typically possess higher levels of education and greater awareness of health and safety.

In two-pollutant models, the associations of IS with PM_2.5_ and NO_2_ remained significant when adjusting for CO. However, with the addition of NO_2_, the estimated effect of CO was inversely correlated. This phenomenon aligns with previous research examining emergency room visits due to kidney disease concerning ambient air pollution in Korea [21]. A study have suggested that exposure to motor traffic (NO_2_ and CO) affected the incidence of stroke [22]. Kim, S found that both short- and long-term exposure to CO were positively related to stroke [23]. However, a study in Hong Kong showed that short-term exposure to ambient CO was associated with decreased risk of emergency hospitalizations for stroke [24]. The short-term beneficial effects of ambient CO on stroke onset are biologically plausible. Recent experimental and clinical studies have demonstrated that the protective effects of CO in ischemic stroke are mediated by the upregulation of nuclear factor-erythroid 2-related factor 2 (Nrf2). In the context of ischemic stroke, CO exerts its protective role through the upregulation of Nrf2, a transcription factor that binds to response elements in the promoter region of the heme oxygenase-1 (HO-1) gene [25]. In summary, previous population-based epidemiological studies examining the relationship between environmentally related CO exposure and stroke hospitalization have produced mixed results.

The impact of ambient air pollutants on hospital admissions for IS in Inner Mongolia was found to be statistically significant at certain lag structures. Research indicated that the effects of NO_2_ and CO were more pronounced with short-term exposure, aligning with previous findings by Phosri A [26]. Similarly, Andersen Z et al. observed a positive association between IS admissions and NO_x_ and CO at lag 4 in Copenhagen between 2003 and 2006 [27]. On the other hand, prolonged exposure to PM seemed to pose a higher cardiovascular risk compared to short-term exposure [28]. Some studies suggest that long-term exposure to air pollutants may contribute to stroke incidence, as seen in research by Wolf K et al. involving six European cohorts even below standard pollutant concentrations [29]. Contrary to these findings, a study in Thailand indicated that O_3_ had no significant impact on hospital admissions for cardiovascular diseases [26]. Similarly, a study in San Jose dos Campos, Sao Paulo, Brazil by Nascimento, LF et al. concluded that O_3_ was not correlated with stroke hospitalization rates [30]. Nevertheless, there are differing perspectives in some studies. Wang, Z and colleagues demonstrated that low O_3_ concentrations could elevate the risk of cerebral infarction in a non-linear and lagging manner [31]. Furthermore, sex differences in various cardiovascular diseases and biological processes at different levels are widely recognized. Men and women also exhibit differing susceptibilities to air pollution exposure concerning various cardiovascular endpoints. The differential responses to air pollution-related cardiovascular diseases in men and women may be attributed to variations in hormones, body structure (e.g., respiratory tract differences between the sexes), metabolism, and social behaviors. Regarding young people, the incidence of cardiovascular and cerebrovascular diseases is higher in the northern regions compared to the southern regions, particularly concerning stroke. Firstly, individuals from the north tend to exhibit a relatively bad temper and a more impatient personality. Secondly, they are more likely to consume a high-salt diet. These two factors are particularly prevalent among young people. Additionally, the colder climate in the north may further contribute to the increased incidence of cardiovascular and cerebrovascular diseases [32].

Previous studies have shown that age and sex are important factors in the impact of air pollution on stroke. Consistent with other research, it was found that the risk of hospital admissions for IS was higher in males. In single-pollutant models, particulate matter was shown to have a significant effect on IS admissions in males [33, 34]. Additionally, in cumulative lag models, carbon monoxide (CO) was found to be significantly correlated with stroke admissions in males. Interestingly, in cumulative lag models, neither of the air pollutants were associated with stroke admissions in females. It is important to note that two studies conducted in southern China reported conflicting results [35, 36].

Our study has several notable advantages. First, while numerous previous studies have established an association between ischemic stroke and air pollutants, most have been conducted in economically developed regions, leaving a gap in the literature regarding research in Inner Mongolia. Secondly, this study specifically targets ethnic minorities, which enhances its relevance and can inform the prevention and control of air pollutants as well as the development of public health strategies in Inner Mongolia. Finally, we employed a longer time frame to assess the cumulative effects of air pollutants, rather than focusing solely on short-term impacts. This approach yields results that differ from analyses based on 3–4 day periods, thereby highlighting that the detrimental effects of air pollutants represent a long-term and evolving concern.

This study holds considerable significance for the formulation of public health strategies in Inner Mongolia. Notably, the findings indicate that the lag effect of air pollutants is more prolonged than reported in other studies, and there are marked gender differences in the cumulative effects of air pollutants, excluding ozone. Consequently, it is crucial to allocate resources effectively, establish a long-term control mechanism, and implement a dynamic monitoring system that prioritizes men’s exposure to air pollutants.

The present study also has some limitations. Firstly, hospital outpatients were excluded from our studies, and the strength of the association with air pollution may differ between inpatients and outpatients. Secondly, similar to other ecological studies, we only utilized the daily average monitoring results from one station as proxies for personal exposure levels to ambient air pollution. The use of ambient rather than personal exposure measures is expected to result in exposure misclassification. However, this misclassification is expected to lead one to underestimate the relative risk [1]. Thirdly, our dataset is restricted to a one-year time period, indicating that longer observational studies will be crucial for further exploring the relationships identified in this research. Fourthly, while our study underscores the association between air pollution and ischemic stroke (IS) in a region with a significant proportion of ethnic minorities, we faced limitations in collecting data on race/ethnicity and lifestyle factors (e.g., smoking, diet, occupational exposures) due to restrictions in the hospital’s electronic records. The absence of these measured confounders may affect susceptibility to air pollution and stroke risk, potentially introducing bias into our estimates. Future research should aim to incorporate individual-level data to address these limitations. Additionally, multi-year data are necessary to more thoroughly evaluate the temporal dynamics of the impact of air pollution on IS. Last, the data of hospital admissions we collected was limited in just one hospital in Inner Mongolia, China. Compared to other large cohort studies, this study has a shorter time span and a narrower spatial distribution. Consequently, the results may be biased towards the characteristics of specific prefecture-level cities in Inner Mongolia, potentially failing to accurately represent the overall situation of the entire Inner Mongolia region or the broader population. Additionally, variations in medical resources and service levels, as well as regional and cultural factors and prefecture-level city policies, may further contribute to an insufficient representation of data samples from a single hospital. These limitations are anticipated to be addressed and improved upon in future research. Instead of fully representing the overall situation of Inner Mongolia, Collecting air pollutant data from various meteorological stations in Inner Mongolia, along with data from ischemic stroke patients admitted to multiple hospitals, and conducting spatial modeling or follow-up surveys on personal exposure, can significantly enhance the scientific rigor and quality of the research by providing true and reliable environmental exposure levels.

## Abbreviations

IS: Ischemic stroke
GBD: The Global Burden of Disease Study
CVD: Cardiovascular diseases
DALYs: Disability-adjusted life years
PM_2.5_: Particulate matter with aerodynamic diameter less than 2.5 µm
PM_10_: Particulate matter with aerodynamic diameter less than 10 µm
NO_2_: Nitrogen dioxide
SO_2_: Sulfur dioxide
CO: Carbon monoxide
O_3_: Ozone
OR: Odds ratio
HR: Hazard ratio
ER: excess risk
RR: relative risk
CI: Confidence interval
RR: Risk ratio
NOx: Nitrogen oxides
R^2^: R-Squared
IQR: Interquartile range
SD: Standard deviation
SE: Standard error
HO-1: heme oxygenase-1
Nrf2: nuclear factor-erythroid 2-related factor

## Declarations

### Availability of data and materials

Data described in the manuscript, code book, and analytic code will be made available upon reasonable request.
